# Scorpion Venom Neurotoxins: Molecular Diversity, Mechanisms, and Drug Scaffolds

**DOI:** 10.3390/toxins18010025

**Published:** 2026-01-01

**Authors:** Yun Huang, Peter Muiruri Kamau, Jiamin Wang, Mingyue Gao, Bowen Li

**Affiliations:** 1 State Key Laboratory of Herbage Improvement and Grassland Agro-Ecosystem, College of Ecology, Lanzhou University, Lanzhou 730000, China; 2 Department of Clinical Neurosciences, Hotchkiss Brain Institute, Cumming School of Medicine, University of Calgary, Calgary, AB T2N 4N1, Canada

**Keywords:** scorpion, venom, neurotoxin, ion channel, drug discovery

## Abstract

Venom is a key evolutionary innovation of venomous organisms in the long-term process of survival adaptation. As one of the oldest arthropods, scorpions produce venom rich in bioactive peptides that also constitute a valuable pharmacological resource. Omics-driven discovery and structural biology have expanded the peptide catalog and clarified structure–function principles across disulfide-bridged (DBPs) and non-disulfide-bridged peptides (NDBPs). Within this arsenal, ion-channel targeting neurotoxins predominantly modulate Nav, Kv, Calcium, Chloride, and TRP channels to achieve predation, defense, and competition. Owing to their unique mechanisms of action and significant therapeutic potential, scorpion venom peptides have attracted sustained interest as leads and scaffolds for drug development. This review synthesizes current knowledge of scorpion venom composition, with an emphasis on the pivotal role of neurotoxins, covering their molecular diversity, structural features, and modes of ion-channel modulation, as well as emerging applications in disease treatment.

## 1. Introduction

“Survival of the fittest,” a central tenet of Darwin’s theory of evolution, holds that under constraints of space and resources, individuals with advantageous heritable traits are more likely to survive and reproduce [1]. Venomous animals span both vertebrate and invertebrate lineages and occupy diverse ecological niches [2,3], deploying toxins to subdue prey, deter predators, and mediate intra- and interspecific competition [4,5,6]. Under sustained natural selection, most venom systems have repeatedly evolved into powerful biochemical arsenals, representing a striking case of convergent evolution [7,8,9,10].

Scorpions, ancient arthropods with an evolutionary history of over 400 million years, are among the earliest fully terrestrialized animal lineages [11,12]. The oldest known scorpion taxa include *Dolichophonus loudonensis* and *Eramoscorpius brucensis*, whose fossil records date back to the Silurian period (approximately 430 million years ago), providing critical evidence for understanding early scorpion morphology and the evolutionary transition toward a fully terrestrial lifestyle [13,14,15]. Unlike insects and spiders, which underwent extensive morphological diversification, scorpions have largely retained key ancestral features such as venom glands, book lungs, and pectines, and they have adapted to extreme and fluctuating environments, earning them the moniker “living fossils” [11,16,17,18]. Fossil evidence provides some of the earliest records of scorpions, and anatomical interpretations suggest that a venom apparatus emerged early in their evolutionary history [12,15,17]. Scorpion venom is a complex mixture of bioactive molecules, including neurotoxins, proteases, protease inhibitors, mucopolysaccharides, free amino acids, and inorganic ions [19,20]. This biochemical repertoire underpins both predation and defense, facilitating survival across diverse and often hostile habitats.

To date, approximately 2900 scorpion species have been described, with distributions across all continents except Antarctica [21]. Scorpion envenomation is a widespread public health concern, particularly in tropical and subtropical regions such as the Middle East, North Africa, southern India, Mexico, and Brazil [22,23]. Globally, an estimated 1.2 million stings occur each year [11,24,25]. Most cases cause only local pain and swelling [26]; however, approximately 30% reportedly progress to severe systemic symptoms, including fever, vomiting, convulsions, abnormalities in blood pressure and heart rate, and respiratory distress, and, in rare cases, coma or death [24,25]. Nearly all fatal stings are attributed to species in the family Buthidae [27,28], which are generally more toxic than members of Scorpionidae or Hemiscorpiidae [28,29].

The principal bioactive components responsible for scorpion venom toxicity are small peptides, typically 20–70 amino acids in length [30], that target multiple physiological receptors [27,30]. Among these, neurotoxins that modulate ion channels account for most envenomation symptoms. The first characterized scorpion neurotoxin, AaH I, was purified from the venom of *Androctonus australis* in 1964 and acts on mammalian voltage-gated sodium channels as an α-NaTx [31]. Since then, scorpion venoms have yielded a wide variety of peptides targeting voltage-gated sodium (Nav), potassium (Kv), calcium (Cav), chloride (ClC) channels; acid-sensing ion channels (ASICs), and transient receptor potential (TRP) channels [26,32,33,34]. These toxins modulate ion-channel function by shifting the voltage dependence, altering activation and inactivation kinetics, and changing conductance and open probability [35,36]. Ion channels are essential for neural signaling, muscle contraction, cardiac rhythm, endocrine secretion, and immune responses [37,38,39,40], and their dysfunction is associated with neurological and cardiovascular disorders [41,42], autoimmune and inflammatory conditions [43,44,45], and several cancers [46,47]. Owing to their potency and high selectivity for defined ion-channel isoforms and other receptors, scorpion neurotoxins serve as powerful molecular probes and promising leads or scaffolds for therapeutic design [48,49,50].

Advances in genomic and transcriptomic sequencing, high-resolution mass spectrometry, and structural biology have driven venom research into an era of molecular precision, enabling systematic discovery, annotation, and structure–function analysis of neurotoxin peptides [32,51,52,53,54,55,56,57,58]. To date, numerous scorpion neurotoxins have been identified and functionally characterized [50,59,60], which not only support the development of effective clinical treatments for envenomation, but also provide potent probes and regulators for ion-channel pharmacology [50,61]. This review synthesizes current knowledge of scorpion venom composition and diversity, highlights the molecular mechanisms and pharmacological functions of key neurotoxins, and evaluates their potential as therapeutic scaffolds. These insights lay a foundation for the efficient utilization and transformation of scorpion venom peptides in drug discovery.

## 2. Diversity of Scorpion Venom Components

Scorpion venom is a complex biochemical mixture composed of diverse constituents. In addition to non-protein components such as lipids, free amino acids, nucleotides, and inorganic ions, the biologically active fraction is dominated by small peptides and enzymes [55]. Disulfide-bridged peptides (DBPs) and non-disulfide-bridged peptides (NDBPs) constitute major components of scorpion venom peptides (SVPs) [50,62,63]. NDBPs are typically small peptides consisting of 13–56 amino acids, lacking disulfide bonds, and often showing no precise conserved sequence–function relationship [64,65]. They frequently exhibit multiple bioactivities without a single defined molecular target and have attracted increasing research interest for their broad pharmacological potential, including antimicrobial, antiviral, anticancer, and immunomodulatory effects [66,67]. DBPs usually comprise 30–70 amino acids and are stabilized by disulfide bridges that maintain compact tertiary structures [30]. Many DBPs act as highly specific and potent ion-channel modulators. The well-defined structure–function relationships make DBPs valuable templates for drug discovery and molecular engineering [68].

## 3. Non-Disulfide-Bridged Peptides

**Figure 1:**
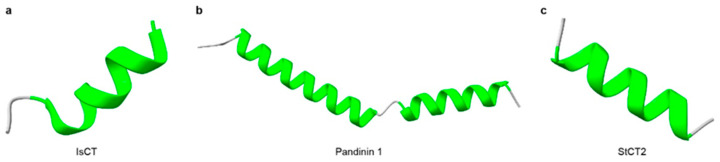
Three-dimensional structures of representative scorpion non-disulfide-bridged peptides (NDBPs). (**a**) The structure of Class I NDBP (IsCT). (**b**) The structure of Class II NDBP (Pandinin 1). (**c**) The structure of Class III NDBP (StCT2). α-Helical regions are highlighted in green. The structure of IsCT was obtained from RCSB PDB (PDB: 1T51), the structures of Pandinin 1 and StCT2 were predicted with AlphaFold3.

Non-disulfide-bridged peptides (NDBPs) constitute more than one-third of known scorpion venom peptides [64,69,70]. They are typically short peptides of 13–56 amino acids that usually lack cysteines and display marked sequence and functional diversity [64] (Table 1). Most NDBPs bear C-terminal amidation (often on Lys, Arg, or His), which confers overall cationicity and promotes interaction with negatively charged biological membranes [71,72]. Structurally, NDBPs are predominantly α-helical peptides and could be grouped into three classes based on the distribution of α-helices along the backbone [64]. Class I comprises a single α-helix flanked by disordered coil regions at both termini; representatives include HsAP, BmKb1, Pandinin 2, BmKn2, IsCT, Meucin-24, Im-1, AamAP1, AamAP2, and Mauriporin [73,74,75,76,77,78,79,80] (Figure 1a). Class II contains two α-helices connected by a flexible coil, such as BmKbpp, Opistoporin 1, Hadrurin, Pandinin 1, and Parabutoporin [80,81,82,83,84] (Figure 1b). Class III consists of peptides that are essentially fully α-helical structures, with only a few identified members, such as StCT2 and Imcroporin [85,86] (Figure 1c). Although NDBPs lack conserved motifs or defined molecular targets, their multifunctional pharmacological activities, particularly antimicrobial, antiviral, and bradykinin-potentiating effects, make them an attractive reservoir of lead molecules [87].

The global rise of multidrug-resistant pathogens has intensified interest in antimicrobial peptides (AMPs) [107], which are small, cationic peptides composed of approximately 15–150 amino acids [108]. Owing to their broad-spectrum activity, short length, structural simplicity, and relatively low propensity to induce resistance, AMPs are promising alternatives to conventional antibiotics. Most scorpion-derived NDBPs are functionally categorized as AMPs. Their positively charged surfaces interact with negatively charged bacterial membranes, disrupt membrane integrity, and form pores that cause leakage of cellular contents and bacterial death [109]. Scorpion AMPs such as Imcroporin, StCT1, Ctriporin, Pantinin 1, Pantinin 2, and Pantinin 3, retain potent activity even against methicillin-resistant *Staphylococcus aureus* (MRSA) and other resistant strains [85,86,110,111]. However, many antimicrobial NDBPs display variable hemolytic and cytotoxic effects. For instance, VmCT2 from *Vaejovis mexicanus* shows 84% hemolysis at 50 μM, whereas VsCT1 from *Vaejovis subcristatus* exhibits only 12% under the same conditions [112]. Therefore, structural optimization and rational peptide design are essential to improve selectivity and safety for clinical applications.

Beyond antimicrobial activity, NDBPs exhibit broad bioactivity profiles. Several scorpion-derived NDBPs show anticancer effects, including Mauriporin, Pantinin, RK1, TsAP-1, TsAP-2, Gonearrestide, AaeAP1a, and AaeAP2a [80,113,114,115,116]. BmKn2 from *Olivierus martensii* (*O. martensii*) induces apoptosis in human oral squamous carcinoma (HSC4) and epidermoid carcinoma (KB) cells through a p53-dependent intrinsic pathway while sparing normal gingival (HGC) and dental pulp (DPC) cells [117]. Some NDBPs, such as Peptide T, Peptide K12, TsHpt, and BmKbpp, show bradykinin-potentiating activity by inhibiting angiotensin-converting enzyme (ACE)-mediated degradation of bradykinin, thereby promoting vasodilation and lower blood-pressure [91,99,101,118,119]. Recent studies also report in vitro antiviral activity against hepatitis C virus (HCV), herpes simplex virus (HSV), and human immunodeficiency virus (HIV) [50]. Examples include Hp1090 and Ctry2459, which inhibit HCV replication [102,103], and Hp1036, Hp1239, Eval418, and Kn2-7, which suppress HSV and HIV [90,104,105]. Moreover, several NDBPs act as immunomodulators, capable of modulating innate and adaptive immune responses through interactions with host cell membranes or immune receptors [120]. Collectively, scorpion-derived NDBPs offer versatile molecular templates for antimicrobial, anticancer, antiviral, and immunotherapeutic development.

## 4. Disulfide-Bridged Peptides

Disulfide-bridged peptides (DBPs) are typically 30–70 amino acids in length and contain three to four intramolecular disulfide bonds, which confer high structural stability and resistance to proteolysis [121]. Most DBPs adopt compact, cystine-stabilized folds that enable precise molecular recognition [122]. Unlike NDBPs, DBPs are usually specific ion channel-acting peptides, including voltage-gated sodium (Nav), potassium (Kv), calcium (Cav), chloride (ClC) channels, and transient receptor potential (TRP) channels [123]. These channel proteins are central to normal cellular physiology [123]. Scorpion DBPs bind their targets with high affinity and selectivity, altering ion permeability or gating characteristics, which may produce neurotoxic, cardiotoxic, or cytotoxic effects [123]. Because of their potency, selectivity, and structural diversity, scorpion DBPs have attracted considerable pharmacological interest and serve as powerful molecular probes for dissecting ion-channel function and as promising leads for the treatment of channelopathies [124].

### 4.1. Toxins Targeting Voltage-Gated Sodium Channels (NaTx)

**Figure 2:**
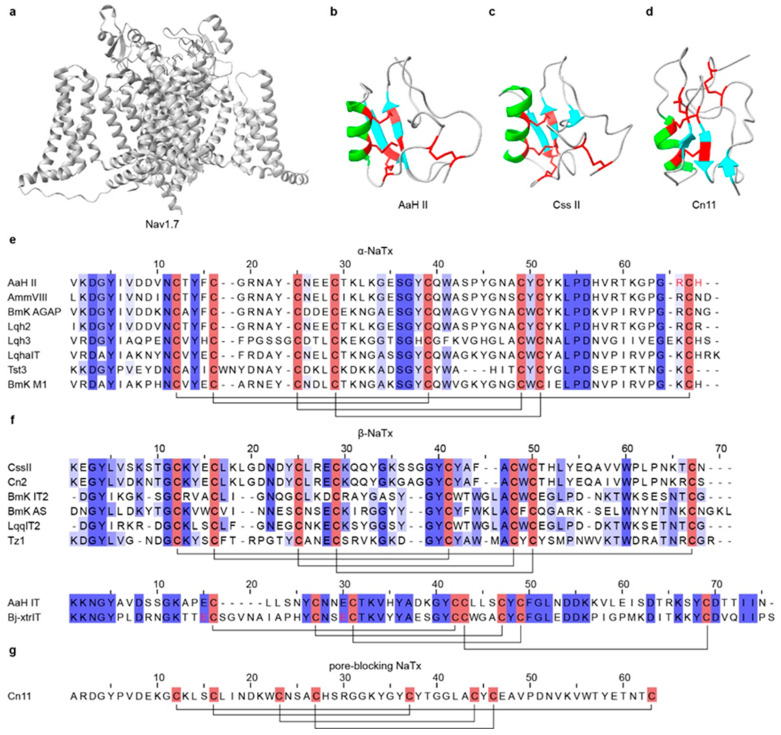
Three-dimensional structures and sequence alignments of representative scorpion NaTx and their target channel. (**a**) The structure of the Nav1.7 channel (PDB: 6J8G). (**b**) The structures of α-NaTx (AaH II, PDB: 1AHO; Ts1, PDB: 1B7D). (**c**) The structures of β-NaTx (Css II, PDB: 2LI7; Cn2, PDB: 1CN2). (**d**) Structure of a pore-blocking NaTx, Cn11 (PDB: 1PE4). (**e**) Multiple sequence alignment of representative α-NaTx. (**f**) Multiple sequence alignment of representative β-NaTx. (**g**) Multiple sequence alignment of representative pore-blocking NaTx. In the three-dimensional structures, α-helices are highlighted in green, β-strands are highlighted in cyan, and disulfide bonds are shown in red. In the sequence alignments, cysteine residues are highlighted in red, disulfide bonds are indicated by black connecting lines, and functionally important amino acid residues are marked in red. All structures were obtained from RCSB PDB.

Voltage-gated sodium channels (Nav) are expressed in most excitable cells and play key roles in the initiation and propagation of action potentials [125] (Figure 2a). Nav channels are primary pharmacological targets for local anesthetics, antiarrhythmics, and antiepileptics [26,126]. Among scorpion venom components, sodium-channel toxins (NaTx) are the principal neurotoxins that act directly on Nav channels, disrupting their normal function and inducing various characteristic envenomation symptoms [26]. NaTxs typically consist of 60–76 amino acids; most contain eight cysteines forming four disulfide bridges that lock an α-helix and two antiparallel β-strands into a compact and stable cysteine-stabilized α/β (CSαβ) fold conformation [125,127,128], which represents the most typical and conserved structural scaffold among scorpion peptide toxins [129,130]. In a minority of NaTx, only three disulfide bonds are present, yet the overall CSαβ topology is retained (Table 2).

For well-characterized NaTxs, classification is typically based on their biophysical effects and experimentally validated binding sites on sodium channels, distinguishing gating modifiers from pore blockers. However, functional classification schemes may overlook some phylogenetically divergent potential NaTxs with specialized functions [32,168]. Gating modifiers NaTx constitute the vast majority and are further divided into α-NaTx and β-NaTx, whereas pore-blocking NaTx are rarely reported [169]. α-NaTx binds to site 3 in domain IV of Nav channels and interfere with the normal movement of the S4 voltage sensor during fast inactivation, thereby suppressing or delaying channel inactivation [170,171,172,173]. The resulting persistent sodium influx drives sustained neuronal firing and can produce symptoms such as pain, muscle spasms, convulsions, and respiratory paralysis [26,174] (Figure 2e). AaH II, an α-NaTx from *Androctonus australis*, is a mammal-specific toxin of 64 residues [175]; it inhibits fast inactivation of Nav1.2 and Nav1.6, prolonging action potentials and evoking intense pain and muscle convulsion [131] (Figure 2b). Lqh-2 shows mammalian-like toxicity by impairing the Nav channel inactivation [155,176,177]. By target preference, α-NaTx are commonly further divided into three subgroups: classical α-toxins (mammalian-preferring), insect α-toxins (insect-preferring), and α-like toxins (active on both mammalian and insect NaV channels) [27,169]. β-NaTx binds to site 4 in domain II of Nav channels [171,178]. Rather than inhibiting inactivation, they interact with the voltage-sensing domain to modify activation kinetics and shift the activation threshold toward more negative potentials, rendering the channels hyperexcitable [179,180] (Figure 2f). CssII, a β-NaTx from *Centruroides suffusus*, specifically targets Nav1.6 by binding to the extracellular end of the domain II voltage sensor, stabilizing an activated conformation and facilitating channel opening [137]. Cn2 from *Centruroides noxius* acts through the same mechanism and shares high sequence similarity with CssII [139] (Figure 2c). In contrast, pore-blocking NaTx are exceedingly rare; Cn11 from *Centruroides noxius* is a well-characterized example proposed to inhibit Nav channels by physically occluding the ion-conducting pore rather than by modifying channel gating [140] (Figure 2d,g).

Given the direct involvement of Nav channels in pain perception and transmission, scorpion-derived Nav modulators are being explored as analgesic leads [181]. Numerous NaTx have been reported to exert analgesic effects by selectively modulating Nav channel subtypes, particularly Nav1.1, Nav1.6, Nav1.7, Nav1.8, and Nav1.9 [182,183]. Several peptides from *O. martensii* exhibit analgesic activity, including BmKBTx [141], BmNaL-3SS2 [141], and DKK-SP2 [142], which specifically target Nav1.7. BmK IT2 [184] and BmK IT-AP [185], display dual bioactivity, being insecticidal and analgesic in mice. Additional Nav channel-targeting peptides with analgesic activity include LqqIT2 from *Leiurus quinquestriatus* [185], AmmVIII from *Androctonus mauretanicus* [185], and BotAF from *Buthus occitanus* [158]. Moreover, studies have shown that voltage-gated sodium channels are often aberrantly upregulated in advanced epithelial cancers, where they contribute to invasion and metastasis [186,187,188]. Thus, Nav inhibitors are being considered for oncology applications. Notably, BmK AGAP from *O. martensii* exhibits both analgesic and anticancer activities; it suppresses the migration and invasion of human breast cancer cells (MDA-MB-231 and MCF-7), and inhibits epithelial–mesenchymal transition (EMT) and the acquisition of stem-like phenotypes [145,189].

### 4.2. Toxins Targeting Voltage-Gated Potassium Channels

**Figure 3:**
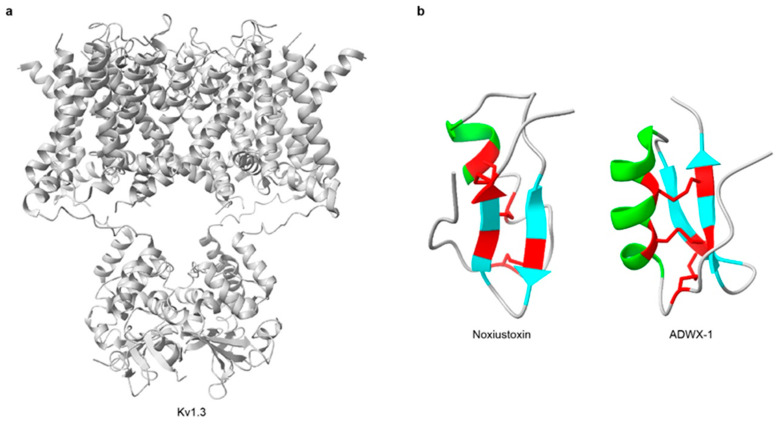
Three-dimensional structures of representative scorpion KTx and their target channel. (**a**) The structure of the Kv1.3 channel (PDB: 7SSX). (**b**) The structures of KTx (Noxiustoxin, PDB: 1SXM; ADWX-1, PDB: 2K4U). In the three-dimensional structures, α-helices are highlighted in green, β-strands are highlighted in cyan, and disulfide bonds are shown in red. All structures were obtained from RCSB PDB.

Voltage-gated potassium channels (Kv) form a large and evolutionarily conserved ion-channel family expressed in virtually all cell types. They shape action potentials in excitable cells, such as neurons, cardiomyocytes, and muscle fibers, in coordination with Nav channels. Kv channels are also involved in the regulation of cell volume, proliferation, and migration in diverse cell types [190,191]. Notably, Kv1.3 is highly expressed in macrophages, microglia, platelets, B cells, T lymphocytes, and the central nervous system (CNS), and is implicated in the pathogenesis and treatment of autoimmune diseases and cancers [192,193] (Figure 3a).

**Figure 4:**
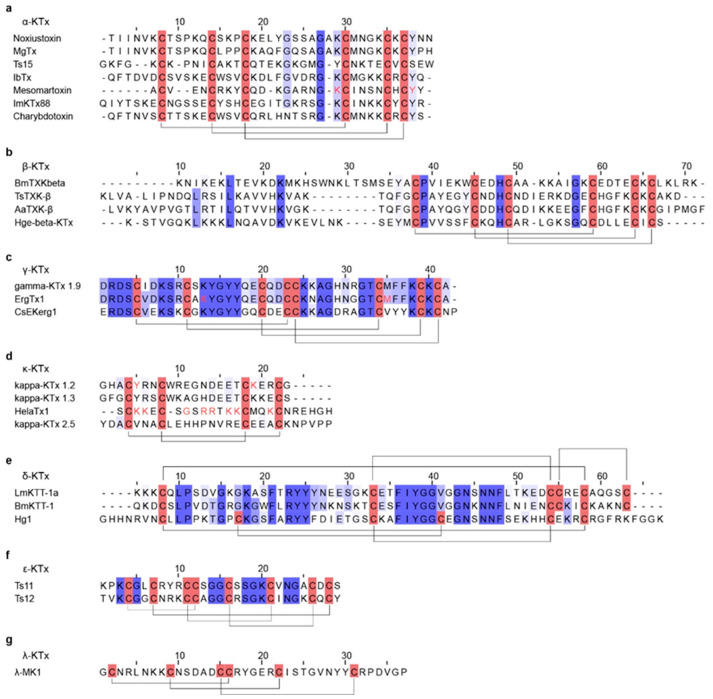
Sequence alignments of representative scorpion KTx. (**a**) Multiple sequence alignment of representative α-KTx. (**b**) Multiple sequence alignment of representative β-KTx. (**c**) Multiple sequence alignment of representative γ-KTx. (**d**) Multiple sequence alignment of representative κ-KTx. (**e**) Multiple sequence alignment of representative δ-KTx. (**f**) Multiple sequence alignment of representative ε-KTx. (**g**) Multiple sequence alignment of representative λ-KTx. In the sequence alignments, cysteine residues are highlighted in red, disulfide bonds are indicated by black connecting lines, and functionally important amino acid residues are marked in red.

Potassium-channel toxins (KTx) are among the most common and extensively studied scorpion neurotoxins [194]. These short peptides typically comprise 23–64 amino acids and contain 2-4 disulfide bridges that form the characteristic cystine-stabilized α/β (CSαβ) fold [124,192,195] (Table 3). Noxiustoxin was the first isolated short-chain scorpion toxin; it specifically reduced K^+^ permeability in the squid giant axon without altering voltage-dependent gating [196,197] (Figure 3b). Since the discovery, more than 199 distinct KTx peptides have been identified in scorpion venoms [50]. For instance, charybdotoxin, purified from *Leiurus quinquestriatus*, is a high-affinity, reversible blocker of Ca^2+^-activated K^+^ channels that occludes the external pore without affecting voltage-dependent gating [198,199]. Based on sequence features, structural motifs, and disulfide-bond patterns, KTx are grouped into: α-KTx, β-KTx, γ-KTx, κ-KTx, δ-KTx, λ-KTx, and ε-KTx [124,200,201] (Figure 4). Among these, α-KTx is the largest and best characterized, with 31 subfamilies currently identified. α-KTx peptides generally contain 23–42 residues, which typically block K^+^ flux either by physically occluding the pore [195] or by interacting with negatively charged residues near the outer vestibule of the channel [202]. It also should be noted, however, that this classification is predominantly based on function and does not necessarily represent actual evolutionary relationships, as phylogenetic analyses have demonstrated that some functionally defined KTx groups do not share a recent common ancestor [203,204].

Several scorpion KTx selectively target the Kv1.3 channel and have shown therapeutic potential in autoimmune disease models [193], including ImKTx88 [231,248], Vm24 [232,233], HsTX1 [249,250], Ts6 [234], Ts15 [234], etc. ADWX-1, a 37-residue derivative of BmKTX, is a highly selective Kv1.3 blocker with remarkable affinity and efficacy [251]. It preferentially inhibits Kv1.3 on CCR7^−^ effector memory T (TEM) cells, suppressing Ca^2+^-dependent activation and proinflammatory cytokine release, thereby effectively alleviating experimental autoimmune encephalomyelitis (EAE) [224]. Ts6 and Ts15 from *Tityus serrulatus* potently inhibit Kv1.3, and Ts15 also blocks Kv2.1. Both peptides reduce T cell-mediated delayed-type hypersensitivity (DTH) responses in vivo [234], highlighting their potential for autoimmune therapy.

Beyond immunomodulation, KTx also exhibit analgesic, antiviral, and anticancer activities. Heterolaxin from *Heterometrus laoticus* and leptucin from *Hemiscorpius lepturus* are representatives with pain-relieving effects [239,246]. Two defensins from *O. martensii*, BmKDfsin3 [252] and BmKDfsin4 [253], exhibit both antimicrobial and Kv1.3-blocking effects. BmKDfsin3 inhibits hepatitis C virus (HCV) infection in a dose-dependent manner by suppressing p38 MAPK activation at noncytotoxic concentrations [226,254]. BmKDfsin4 dose-dependently reduces hepatitis B virus (HBV) DNA and viral protein production [255]. Additional KTx with antiviral activity include Smp76 from *Scorpio maurus*, which acts via an interferon-β (IFN-β) like mechanism to inhibit established infections, with significant inhibitory effects on dengue virus (DENV) and Zika virus (ZIKV) [236,237]. Ev37 from *Euscorpiops validus*, which alkalizes intracellular acidic organelles to prevent low-pH-dependent fusion between viral and endosomal membranes, thereby blocking release of the viral genome into the cytoplasm [256]. Ev37 has been reported to inhibit DENV-2, HCV, ZIKV, and herpes simplex virus type 1 (HSV-1) infections [238]. In oncology, margatoxin (MgTx) and iberiotoxin (IbTx) are promising leads. MgTx, a 39-residue α-KTx with three disulfide bridges, blocks mammalian Kv1.3 and suppresses proliferation of human lung adenocarcinoma A549 cells [257,258]. The large-conductance Ca^2+^-activated K^+^ channel (BK) is widely expressed in glioma and may regulate tumor growth [259,260,261]. IbTx, a BK-specific blocker [262], inhibits proliferation of human 1321N1 astrocytoma cells and induces S-phase arrest [259,263]. Collectively, KTx are rich sources of pharmacological tools and therapeutic leads. Their potency, isoform selectivity, and compact scaffolds support applications in immune modulation, antiviral therapy, pain management, and cancer treatment.

### 4.3. Toxins Targeting Calcium Channels (CaTx)

**Figure 5:**
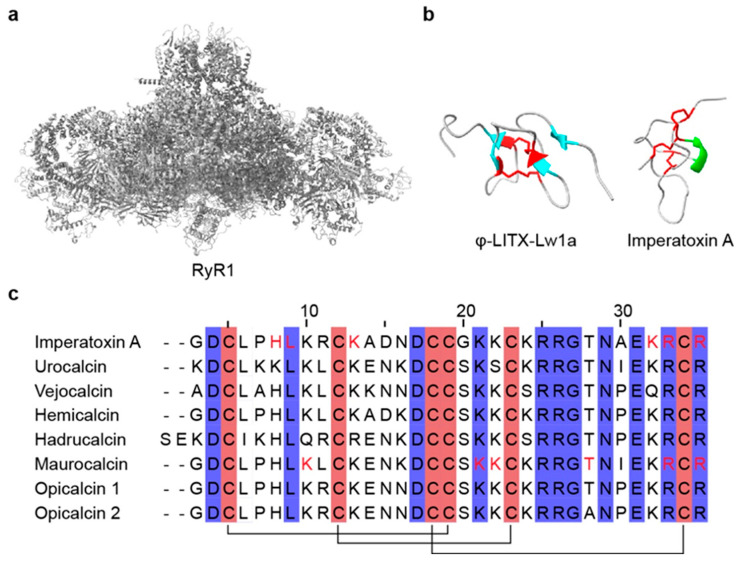
Three-dimensional structures and sequence alignment of representative scorpion CaTx and their intracellular target. (**a**) The structure of the RyR1 channel (PDB: 8SEN). (**b**) The structures of CaTx (φ-LITX-Lw1a, PDB: 2KYJ; Imperatoxin A, PDB: 1IE6). (**c**) Multiple sequence alignment of representative CaTx. In the three-dimensional structures, α-helices are highlighted in green, β-strands are highlighted in cyan, and disulfide bonds are shown in red. In the sequence alignment, cysteine residues are highlighted in red, disulfide bonds are indicated by black connecting lines, and functionally important amino acid residues are marked in red. All structures were obtained from RCSB PDB.

Calcium ions (Ca^2+^) play a key role in numerous physiological processes, including neurotransmission, muscle contraction, hormone secretion, and intracellular signaling [264,265,266]. Ca^2+^ influx across the plasma membrane and release from intracellular stores are mediated by voltage-gated calcium channels (VGCCs) and ryanodine receptors (RyRs), respectively [267,268] (Figure 5a). Among scorpion venom components, a subset of peptides collectively termed CaTx, acts directly or indirectly on these channels, thereby modulating Ca^2+^ permeability and intracellular Ca^2+^ levels [269,270]. Toxins that target calcium channels are valuable probes for basic physiological research and hold significant pharmacological potential. CaTx typically contains 33–36 amino acids and 2-4 disulfide bridges [54,271] (Table 4). Based on structural characteristics, they are grouped into two motifs: the inhibitor cystine knot (ICK) fold and the disulfide-directed hairpin (DDH) fold [272]. Most scorpion CaTx act on intracellular Ca^2+^-release channels (RyRs), whereas only a few target VGCCs [273,274].

Notable VGCC-active examples include kurtoxin and kurtoxin-like I. Kurtoxin, identified from *Pandinus transvaalicus*, shares sequence similarity with NaTx and selectively interacts with VGCCs [274]. It binds the α1G subunit of T-type calcium channels and suppresses activity by altering voltage-dependent gating kinetics [270,275]. Thus, kurtoxin could serve as a molecular tool for studying the functions of T-type calcium channels. Kurtoxin-like I from *Parabuthus granulatus* differs from kurtoxin by six residues yet exhibits similar function on T-type channels [276]. Calcins are small scorpion peptide toxins of approximately 33–35 residues with three disulfide bridges that adopt a stable ICK fold [278] (Figure 5c). They possess positively charged surfaces and can penetrate cell membranes. Calcins specifically target RyRs in the endoplasmic and sarcoplasmic reticulum, where they modulate channel opening to regulate Ca^2+^ release from intracellular stores [282,283]. To date, numerous calcin-like sequences have been described, primarily through transcriptomic and proteomic studies [54,284,285]; however, detailed functional characterization has been reported for only a limited subset, including urocalcin [278], vejocalcin [278], hemicalcin [279], hadrucalcin [273], maurocalcin [286], opicalcin 1 [278], opicalcin 2 [278], imperatoxin A [278,287], and intrepicalcin [277]. Liotoxins are a subclass of CaTx that also act on RyRs but adopt a DDH rather than an ICK fold [50,275]. The peptide ϕ-LITX-Lw1a, the first DDH motif CaTx isolated from scorpion venom, efficiently targets and activates mammalian RyRs [272] (Figure 5b). A key functional difference between calcins and liotoxins is cell permeability: calcins are cell-penetrating due to amphipathic α-helical regions, whereas liotoxins lack such structural features and do not rapidly cross the plasma membrane to reach intracellular RyRs [272].

RyRs are intracellular Ca^2+^ release channels located on endoplasmic and sarcoplasmic reticulum membranes and are essential for Ca^2+^ homeostasis [288]. Among the three isoforms, RyR2 is the principal channel in cardiomyocytes and is critical for excitation–contraction coupling [266,289,290]. Dysfunction of RyR2 has been linked to severe cardiac disorders, including ventricular arrhythmia, heart failure, and sudden cardiac death [291,292]. Consequently, pharmacological modulation of RyR2 is a promising therapeutic strategy. Maurocalcin, a 33-residue peptide toxin, increases RyR2 sensitivity to cytosolic Ca^2+^, thereby enhancing channel opening and regulating Ca^2+^ release in cardiomyocytes [293], which suggests utility in certain types of arrhythmias. Imperatoxin A (IpTxa), from *Pandinus imperator*, is an intracellular RyR1 activator that promotes Ca^2+^ release from the sarcoplasmic reticulum [287] (Figure 5b). Remarkably, IpTxa is cell-penetrant and can deliver otherwise impermeant cargos into cells [294], providing valuable strategies for targeted intracellular delivery.

### 4.4. Toxins Targeting Chloride Channels (ClTx)

Voltage-gated chloride channels (ClCs) are homodimeric proteins that conduct Cl^−^, NO_3_^−^, SCN^−^, and other anions and participate in numerous physiological processes, including cell growth, pH regulation, modulation of cellular excitability, ionic homeostasis, etc [123]. In mammals, ClC-1, ClC-2, and ClC-K are expressed at the plasma membrane, where they stabilize membrane potential and maintain excitability, with essential roles in skeletal muscle relaxation and renal water–salt balance [295,296]. By contrast, ClC-3 through ClC-7 function predominantly as Cl^−^/H^+^ exchangers on intracellular organelle membranes, maintaining organelle ionic homeostasis and acidic environments [297,298].

**Figure 6:**
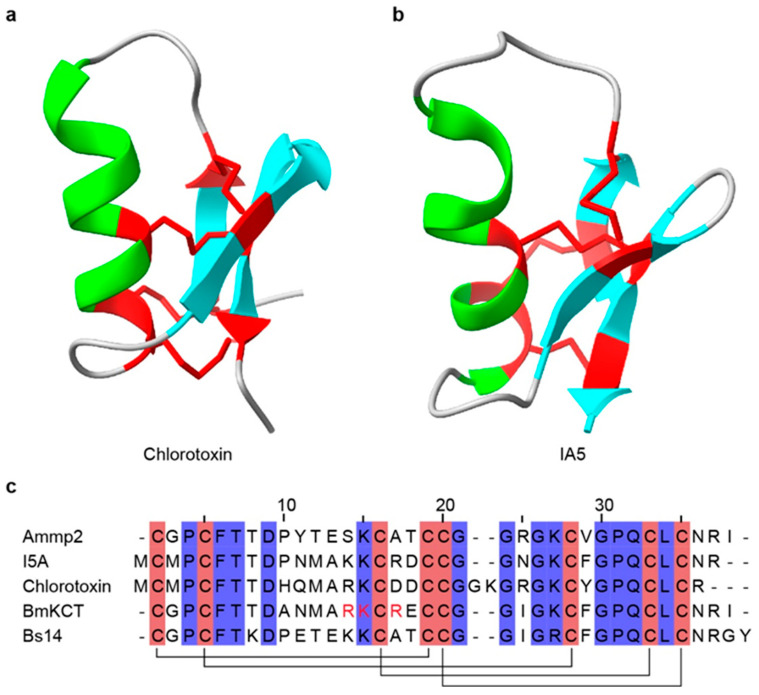
Three-dimensional structures and sequence alignment of representative scorpion ClTx. (**a**) The structure of Chlorotoxin (PDB: 1CHL) and (**b**) I5A (PDB: 1SIS). (**c**) Multiple sequence alignment of representative ClTx. In the three-dimensional structures, α-helices are highlighted in green, β-strands are highlighted in cyan, and disulfide bonds are shown in red. In the sequence alignment, cysteine residues are highlighted in red, disulfide bonds are indicated by black connecting lines, and functionally important amino acid residues are marked in red. All structures were obtained from RCSB PDB.

Currently, only a limited number of scorpion toxin peptides that target chloride channels (ClTx) have been identified, including Ammp2 [299], chlorotoxin [300,301,302] (Figure 6a), I5A [303] (Figure 6b), Lqh7-1 [304], Lqh2-2 [304], Lqh8-6 [304], BmKCT [305], Bs14, and PBITX1 [306,307] (Table 5 and Figure 6c). Among these, chlorotoxin (CTx) is the first to be identified and the best characterized ClTx from *Leiurus quinquestriatus*. CTx is a 36-residue peptide with four disulfide bonds that inhibits Cl^−^ influx across the plasma membrane [308]. CTx shows selectivity for glioblastoma cells, suppressing migration and invasion while exerting minimal effects on normal cells, highlighting its potential as an anticancer molecule [309]. CTx binds matrix metalloproteinase 2 (MMP-2), which is overexpressed in glioblastoma, reducing gelatinase activity in membrane-associated complexes and downregulating MMP-2 expression [302,310]. CTx also blocks ClC-3 channels, inhibiting cell volume changes and reducing migratory speed, and is ultimately endocytosed after binding [311]. Because of its high tumor specificity, biotinylated or fluorescently labeled CTx has been used as a glioma-specific tracer [300]. A homologous peptide, BmKCT from *O. martensii*, consists of 35 residues with four disulfide bonds and similarly binds glioma cells, inhibiting proliferation and migration with minimal effects on normal cells [305]. BmKCT is therefore another promising diagnostic and therapeutic candidate for glioblastoma. Additionally, three short-chain peptides, Lqh2-2, Lqh7-1, and Lqh8-6, were isolated from *Leiurus quinquestratus* [304,312]. Electrophysiology and intracellular Ca^2+^ imaging indicate that these peptides inhibit calcium-activated chloride channel currents in vascular smooth muscle cells, which may be consistent with targeting the TMEM16A/Ano1 channel [304]. These peptides provide useful pharmacological tools for studying calcium-activated chloride channels (CaCCs).

### 4.5. Toxins Targeting TRP Channels

**Figure 7:**
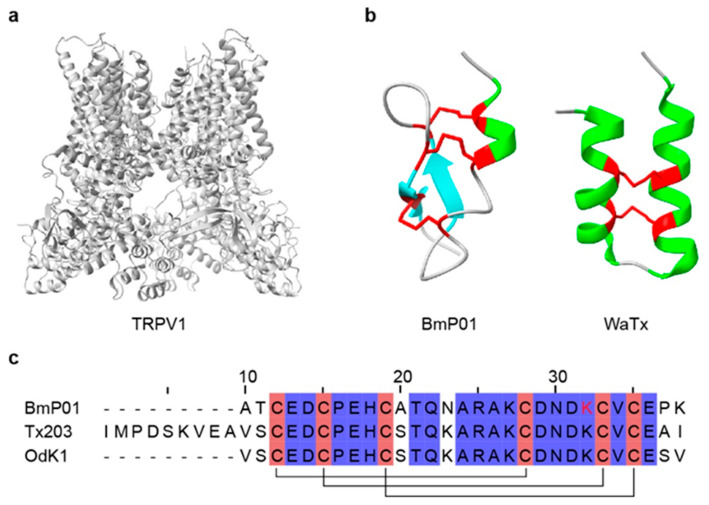
Three-dimensional structures and sequence alignment of representative scorpion toxins targeting TRP channels and their target. (**a**) The structure of the TRPV1 channel (PDB: 9P6B). (**b**) The structures of BmP01 (PDB: 1WM7) and WaTx (PDB: 6OFA). (**c**) Multiple sequence alignment of representative TRPTx. In the three-dimensional structures, α-helices are highlighted in green, β-strands are highlighted in cyan, and disulfide bonds are shown in red. In the sequence alignment, cysteine residues are highlighted in red, disulfide bonds are indicated by black connecting lines, and functionally important amino acid residues are marked in red. All structures were obtained from RCSB PDB.

Transient receptor potential (TRP) channels are nonselective cation channels mainly expressed on the plasma membrane of peripheral and central neurons [314,315,316], and are central to cellular signal transduction, sensory perception, and homeostasis [317,318] (Figure 7a). These receptors can be activated by chemical ligands, and also by physical stimuli, including osmotic stress, mechanical force, temperature, and light [319,320,321]. Scorpion neurotoxins also modulate TRP channels. To date, a limited set of TRP-targeting toxins from scorpions has been described, including BmP01, WaTx, Tx203, and OdK1 [322,323] (Table 6 and Figure 7c). WaTx, isolated from *Urodacus manicatus*, is a cell-penetrant peptide that targets TRPA1 (Figure 7b). WaTx prolongs the channel’s open state while reducing Ca^2+^ permeability, thereby eliciting acute pain and pain hypersensitivity without triggering neurogenic inflammation [324,325]. This pharmacological profile makes WaTx a valuable probe for elucidating TRPA1 function in nociception and neuronal signal transduction [324]. BmP01, isolated from *O. martensii*, activates TRPV1 in an acid-dependent manner via a “one-two” punch mechanism [325] (Figure 7b). Likewise, OdK1 and Tx203, members of the α-KTx8 subfamily, significantly potentiate TRPV1 under acidic conditions [323,326]. Collectively, these TRP-targeting toxins provide distinctive pharmacological tools for probing nociception and thermosensation, and promote the development of therapies for pain and other channel-related disorders.

## 5. Enzymes

Unlike many snake venoms, enzymes in scorpion venom are generally not the primary toxic agents; instead, they play auxiliary roles that facilitate venom diffusion and promote tissue penetration [329]. With advances in omics, diverse enzymes have been identified in scorpion venoms, including phospholipases, hyaluronidases, metalloproteinases, and serine proteases [51,330,331]. Elucidating their structure–function relationships, molecular evolution, and interactions with peptide toxins are important directions for future scorpion venom research.

Metalloproteinases and serine proteases are two major proteolytic enzyme classes in scorpion venom. Metalloproteinases degrade fibrinogen and neuropeptides, thereby contributing to neurotoxicity and inflammation [332,333,334], and promote host tissue damage and degradation of immune defense proteins [335]. Serine proteases help regulate inflammatory responses and coordinate multiple physiological processes [336,337]. Both classes may post-translationally modify venom peptides and facilitate venom spread within prey, thereby amplifying overall toxicity [200,338,339]. Phospholipases hydrolyze ester bonds in phospholipids and, in snake venoms, contribute to predation and defense by disrupting cell membranes and modulating signaling pathways [340,341]. In scorpion venoms, such as those of *Scorpio maurus* and *Anuroctonus phaiodactylus*, phospholipase A2 (PLA2) is the most common type and exhibits multiple toxic effects, including hemolytic, inflammatory, myotoxic, cardiotoxic, and anticoagulant activities [340,342,343]. Hyaluronidases degrade hyaluronic acid in the extracellular matrix and are generally regarded as “spreading factors” that increase tissue permeability [344,345,346,347]. Studies on *Tityus serrulatus* hyaluronidase indicate that it is crucial for venom diffusion from the sting site to the bloodstream and for biodistribution to target organs [346].

## 6. Non-Peptidic Small Molecules

In addition to bioactive peptides and enzymes, scorpion venom contains a range of non-peptidic small molecules, although research on these constituents remains limited [348]. Studies on snake and spider venoms have demonstrated that small molecules contribute substantially to toxicity and physiological regulation, suggesting similar roles in scorpions [349,350]. Systematic identification and functional characterization of these compounds would clarify the overall mechanisms of venom action and inform improved prevention and clinical management of scorpion envenomation. To date, only a few have been characterized [348]. Examples include an alkaloid from *Megacormus gertschi* [351]; two antibacterial 1,4-benzoquinone derivatives from *Diplocentrus melici* [352]; adenosine with anticoagulant activity in *Heterometrus laoticus* [353,354]; and citrate in *Centruroides sculpturatus* [355]. Analysis of *Heterometrus waigiensis* venom further revealed several small molecules, including adenosine, AMP, citrate, glutamic acid, and aspartic acid [348]. In addition, scorpion venoms contain other small-molecule components, such as nucleotides, lipids, amines, heterocyclic compounds, and inorganic salts [20,75].

## 7. Discussion

Scorpion venom is an evolutionarily refined biochemical arsenal rich in bioactive molecules with distinct pharmacological profiles. Among these, neurotoxins stand out for their selectivity, potency, and structural stability. They act with high selectivity on ion channels or other membrane receptors, thereby modulating a range of physiological processes, including neural transmission, immune response, tumor progression, and pain perception [50,356]. Accordingly, they may serve as precise molecular probes for studying ion-channel function and as templates for rational therapeutic design [61]. Although only a limited number of venom-derived peptide drugs have reached development or clinical use [62], notable examples include Captopril, the first blood pressure medication developed through structural modification of a snake venom peptide [357,358]; Ziconotide, a cone snail peptide approved for the treatment of chronic pain [359]; Eptifibatide, a snake venom-derived antiplatelet drug used in cardiovascular interventions [360,361]. Clinical translation of scorpion peptides is still in its early stages. Chlorotoxin, initially isolated from *Leiurus quinquestriatus*, is the only scorpion peptide that has advanced to clinical trials to date [308].

Despite ongoing venom research identifying numerous peptides with promising in vitro pharmacological properties, clinical translation is constrained by conventional validation processes that typically verify target engagement without accounting for in vivo limitations such as rapid proteolysis, short half-life, inadequate tissue penetration, and off-target liabilities [362,363,364]. Bridging this gap may require developability prioritization and peptides engineering. Artificial intelligence and bioinformatics can mine omics-scale venom resources, improve toxin annotation, and rank candidates by integrating predicted potency with sequence features linked to stability and toxicity, while interpretable models can highlight residues and motifs that guide rational redesign [365,366,367]. These optimized scaffolds can then be strengthened through chemical strategies such as sequence minimization, incorporation of noncanonical residues, cyclization or stapling, and disulfide surrogates, as well as polymer or glycan conjugation when appropriate, to improve conformation, protease resistance, and pharmacokinetics while preserving activity [368,369,370,371]. In parallel, precision nanocarriers and peptide-mediated delivery systems can protect peptides in circulation, tune biodistribution, and enable intracellular delivery, thereby converting strong in vitro bioactivity into therapeutic exposure in vivo [372,373]. Collectively, combining computational design, structural stabilization, and delivery engineering can shift venom peptide research toward better candidates explicitly optimized for translational performance.

Integrative investigations encompassing genomics, transcriptomics, proteomics, and structure biology have uncovered considerable molecular diversity and functional intricacy driven by evolutionary adaptation, thereby enhancing our comprehension of venom biology [11,51,54,55]. High-quality scorpion genomes, together with deep RNA sequencing of venom glands, now enable the comprehensive recovery of expressed toxin repertoires and place them in an explicit evolutionary framework [11,374,375]. Transcriptomics not only increases the number of discovered peptides, but also reveals extensive sequence diversity within toxin families, including low-abundance paralogs and lineage variants [55,285,330,376]. It allows for studying toxin function by analyzing sequence diversity and evolutionary relationships, which is essential for understanding peptide scaffolding [32,69]. Additionally, comparative analyses clarify peptide homology across taxa, highlight scaffold conservation versus hypervariable functional regions, and help distinguish convergent functional recruitment from true shared ancestry, thereby providing a rational basis for selecting peptide scaffolds with stability and target selectivity [329,377]. Proteomics adds an indispensable orthogonal layer by confirming which transcripts are translated into secreted venom components, resolving mature peptide processing and post-translational modifications, and prioritizing candidates with both biological activity and technical operation [51,54,285,330]. When coupled with structural determination and functional assays, this multi omics framework links toxin evolution to mechanism and ultimately to drug scaffold optimization.

Future progress will benefit from discovery-to-development pipelines. High-throughput omics and high-resolution structure determination can accelerate target validation and mechanism elucidation [378,379]. AI-assisted design and peptidomimetic strategies can improve stability, selectivity, and pharmacokinetics [380,381]. Chemical modification and sequence minimization, backbone cyclization, and disulfide surrogates can reduce proteolysis and immunogenicity while preserving activity [382,383]. Formulation with nanocarriers or conjugation to tumor-homing ligands can enhance delivery and tissue selectivity [384,385]. With continued advances, research on scorpion peptides is entering a phase of molecular precision and bioengineering. Scorpion-derived peptides are expected to yield candidates with high specificity and low toxicity for applications in ion-channel modulation, oncology, antiviral therapy, and immune regulation.

## Figures and Tables

**Table 1:** Representative scorpion non-disulfide-bridged peptides (NDBPs).

NDBPs	Scorpion Species	Peptide Length	Biological Activities	Target (MIC)	References
BmKb1	*Olivierus martensii* *	18	Antimicrobial	*Staphylococcus aureus* (8.37 μM) *Escherichia coli* (9.47 μM)	[74,88]
BmKn2	*Olivierus martensii*	13	Antimicrobial, hemolytic	EV71 (7.11 μM) *Staphylococcus aureus* (0.43 μM) *Escherichia coli* (1.07 μM)	[74,88,89]
Meucin-24	*Mesobuthus eupeus*	24	Antimalarial	*Plasmodium berghei* (5.45 μM)	[76]
Meucin-25	*Mesobuthus eupeus*	25	Antimalarial	*Plasmodium berghei* (4.85 μM)	[76]
Kn2-7	*Olivierus martensii*	13	Antiviral	HIV-1 (7.11 μM)	[90]
BmKbpp	*Olivierus martensii*	47	Bradykinin-potentiating	*Staphylococcus aureus* (>70 μM) *Escherichia coli* (2.3 μM)	[91]
IsCT	*Opisthacanthus madagascariensis*	13	Antimicrobial, hemolytic	*Staphylococcus aureus* (30.96 μM) *Escherichia coli* (30.96 μM)	[92]
Opistoporin 1	*Opistophtalmus carinatus*	44	Antimicrobial, Immune-modulatory, hemolytic	*Staphylococcus aureus* (>50 µM) *Escherichia coli* (12.5 µM)	[81]
Im-1	*Isometrus maculatus*	56	Antimicrobial, hemolytic, cytolytic	*Staphylococcus aureus* (13–25 μM) *Escherichia coli* (0.4–0.8 μM)	[78]
Imcroporin	*Isometrus maculates*	17	Antimicrobial, hemolytic, cytolytic	*Staphylococcus aureus* (11.35 μM)	[86]
Mauriporin	*Androctonus mauritanicus*	48	Anticancer, antimicrobial	*Escherichia coli* (1.39 μM)	[93,94]
AamAP1	*Androctonus amoreuxi*	18	Antimicrobial, hemolytic	*Staphylococcus aureus* (20 μM) *Escherichia coli* (150 μM)	[77]
AamAP2	*Androctonus amoreuxi*	18	Antimicrobial, hemolytic	*Staphylococcus aureus* (48 μM) *Escherichia coli* (120 μM)	[77]
Hadrurin	*Hadrurus aztecus*	41	Antimicrobial	*Escherichia coli* (<10 µM)	[82]
Parabutoporin	*Parabuthus schlechteri*	45	Antimicrobial, immune-modulatory, hemolytic	*Staphylococcus aureus* (>50 µM) *Escherichia coli* (3.1 µM)	[81]
Pandinin 1	*Pandinus imperator*	44	Antimicrobial, hemolytic	*Staphylococcus aureus* (2.6 µM) *Escherichia coli* (20.8 µM)	[83]
Pandinin 2	*Pandinus imperator*	24	Antimicrobial, hemolytic	*Staphylococcus aureus* (2.4 µM) *Escherichia coli* (19.1 µM)	[83]
StCT1	*Scorpiops tibetanus*	14	Antimicrobial	*Staphylococcus aureus* (8.06 μM) *Escherichia coli* (>64.48 μM)	[95]
Smp24	*Scorpio maurus*	24	Antitumor, antimicrobial	*Staphylococcus aureus* (3.10 μM) *Escherichia coli* (12.41 μM) HepG2 (5.52 µM) A549 (4.06 µM)	[96,97,98]
Peptide T	*Tityus serrulatus*	13	Bradykinin-potentiating	NA	[99]
TsHpt	*Tityus serrulatus*	25	Bradykinin-potentiating	NA	[100]
Peptide K12	*Buthus occitanus*	21	Bradykinin-potentiating	NA	[101]
Ctry2459	*Chaerilus tryznai*	9	Antiviral	HCV (18.21 μM)	[102]
Hp1090	*Heterometrus petersii*	13	Antiviral	HCV (5.0 μM)	[103]
Hp1036	*Heterometrus petersii*	13	Antiviral	HSV-1 (0.43 μM)	[104]
Hp1239	*Heterometrus petersii*	13	Antiviral	HSV-1 (0.41 μM)	[104]
HsAP	*Heterometrus spinifer*	29	Antimicrobial, hemolytic	*Staphylococcus aureus* (23.6 μM) *Escherichia coli* (51.2 μM)	[79]
Eval418	*Euscorpiops validus*	13	Antiviral	HSV-1 (1.62 μM)	[105]

* *Olivierus martensii* (*O. martensii*), formerly *Mesobuthus martensii* [106].

**Table 2:** Representative scorpion toxins targeting sodium channels (NaTx).

NaTx	Scorpion Species	Peptide Length (S–S Bridge)	Targets	References
AaH II	*Androctonus australis*	64 (4)	Nav1.2, Nav1.6	[131]
AaH IT	*Androctonus australis*	70 (4)	NA	[132]
Acra3	*Androctonus crassicauda*	66 (4)	NA	[133,134]
AmmVIII	*Androctonus mauretanicus*	65 (4)	Nav1.3	[135]
Phaiodotoxin	*Anuroctonus phaiodactylus*	72 (4)	NA	[136]
CssII	*Centruroides suffusus*	66 (4)	Nav1.5, Nav1.6	[137,138]
Cn2	*Centruroides noxius*	66 (4)	Nav1.6	[139]
Cn11	*Centruroides noxius*	63 (4)	NA	[140]
BmKBTx	*Olivierus martensii*	58 (4)	Nav1.7	[66]
BmNaL-3SS2	*Olivierus martensii*	64 (3)	Nav1.7	[141]
DKK-SP2	*Olivierus martensii*	66 (4)	Nav1.7	[142]
BmK IT2	*Olivierus martensii*	61 (4)	Nav1.3, Nav1.8, Nav1.9	[143]
BmKM9	*Olivierus martensii*	64 (4)	Nav1.4, Nav1.5, Nav1.7	[144]
BmK AGAP	*Olivierus martensii*	66 (4)	Nav1.4, Nav1.5, Nav1.7, Nav1.8	[145]
BmK M1	*Olivierus martensii*	64 (4)	Nav1.2, Nav1.6	[146,147,148,149]
BmK AS	*Olivierus martensii*	66 (4)	Nav1.2, Nav1.3, Nav1.8, Nav1.9	[150,151,152]
Syb-prII	*Olivierus martensii*	62 (4)	Nav1.8	[153]
LqqIT2	*Leiurus quinquestriatus*	61 (4)	Nav1.2	[154]
Lqh2	*Leiurus quinquestriatus*	64 (4)	NA	[155]
Lqh3	*Leiurus quinquestriatus*	67 (4)	NA	[155,156]
LqhaIT	*Leiurus quinquestriatus*	66 (4)	NA	[157]
BotAF	*Buthus occitanus*	64 (4)	N/A	[158]
Hj1a	*Hottentotta jayakari*	66 (4)	Nav1.1	[159]
Hj2a	*Hottentotta jayakari*	64 (4)	Nav1.1	[159]
Bj-xtrIT	*Hottentotta judaicus*	76 (4)	NA	[160,161,162]
TsNTxP	*Tityus serrulatus*	63 (4)	Glutamate release	[163]
Ts1	*Tityus serrulatus*	61 (4)	Nav1.4	[164]
Tst3	*Tityus stigmurus*	62 (4)	Nav1.2, Nav1.5, Nav1.7	[165]
Tz1	*Tityus zulianus*	64 (4)	Nav1.4	[166,167]

**Table 3:** Representative scorpion toxins targeting potassium channels (KTx).

KTx	Scorpion Species	Peptide Length (S–S Bridge)	Targets	References
AaTXK-β	*Androctonus australis*	62 (3)	Kv7.4	[205]
Noxiustoxin	*Centruroides noxius*	39 (3)	Kv1.2, Kv1.3	[196]
ErgTx1	*Centruroides noxius*	42 (4)	Kv11.1, Kv11.3, Kv11.2	[206,207,208,209]
MgTx	*Centruroides margaritatus*	39 (3)	Kv1.3	[210,211]
CsEKerg1	*Centruroides sculpturatus*	43 (4)	Kv11	[212]
gamma-KTx 1.9	*Centruroides tecomanus*	42 (4)	Kv11.1	[213]
kappa-KTx 1.2	*Chersonesometrus fulvipes*	23 (2)	Kv10.1, Kv1.2, Kv1.3, Kv1.6	[214,215,216]
Charybdotoxin	*Leiurus hebraeus*	37 (3)	BK	[199,217]
LmKTT-1a	*Lychas mucronatus*	59 (3)	Kv1.3, Kv1.1, Kv1.2	[218,219]
kappa-KTx 2.5	*Opisthacanthus cayaporum*	28 (2)	Kv1.1, Kv1.4	[220]
λ-MK1	*Mesobuthus eupeus*	37 (3)	Shaker	[221]
BeKm-1	*Mesobuthus eupeus*	36 (3)	Kv11.1, Kv11.2, Kv11.3	[208,222,223]
ADWX-1	*Olivierus martensii*	37 (3)	Kv1.3	[224,225]
BmKDfsin3	*Olivierus martensii*	38 (3)	Kv1.3	[226]
BmKDfsin4	*Olivierus martensii*	37 (3)	Kv1.3	[227]
Mesomartoxin	*Olivierus martensii*	29 (3)	Kv1.2	[228]
Martentoxin	*Olivierus martensii*	37 (3)	BK (α + β4)	[229]
BmTXKbeta	*Olivierus martensii*	61 (3)	NA	[230]
BmKTT-1	*Olivierus martensii*	59 (3)	Kv1.3	[219]
ImKTx88	*Isometrus maculates*	39 (3)	Kv1.3	[231]
Vm24	*Vaejovis mexicanus*	36 (4)	Kv1.3	[232,233]
Ts6	*Tityus serrulatus*	40 (4)	Kv1.3	[234]
Ts11	*Tityus serrulatus*	29 (4)	Kv1.2, Kv1.3, Kv4.2, Kv10.1, Kv11	[201]
Ts12	*Tityus serrulatus*	29 (4)	Kv1.2, Kv1.3, Kv1.4, Kv11	[201]
Ts15	*Tityus serrulatus*	36 (3)	Kv1.3, Kv2.1	[234]
TsTXK-β	*Tityus serrulatus*	60 (3)	Kv4.2	[235]
Smp76	*Scorpio maurus*	76 (3)	HCV, DENV and ZIKV	[236,237]
Ev37	*Euscorpiops validus*	78 (3)	DENV2, HCV, ZIKV and HSV-1	[238]
Hge-beta-KTx	*Hoffmannihadrurus gertschi*	58 (3)	Kv1.1, Kv1.2, Kv1.3	[33]
Hg1	*Hoffmannihadrurus gertschi*	67 (3)	Kv1.3	[219]
Leptucin	*Hemiscorpius lepturus*	55 (3)	N/A	[239]
IbTx	*Hottentotta tamulus*	37 (3)	BK	[240,241]
HsTX1	*Heterometrus spinifer*	34 (4)	Kv1.3	[242,243]
kappa-KTx 1.3	*Heterometrus spinifer*	23 (2)	Kv10.1	[216]
HelaTx1	*Heterometrus laoticus*	25 (2)	Kv1.1, Kv1.6	[244,245]
Hetlaxin	*Heterometrus laoticus*	34 (4)	Kv1.1, Kv1.3	[246]
KAaH1	*Androctonus australis*	58 (3)	Kv1.1, Kv1.3	[247]

**Table 4:** Representative scorpion toxins targeting calcium channels (CaTx).

CaTx	Scorpion Species	Peptide Length (S–S Bridge)	Targets	References
Kurtoxin	*Parabutus transvaalicus*	63 (4)	Cav3.1, Cav3.2, Cav3.3	[270,275]
Kurtoxin-like I	*Parabuthus granulatus*	62 (4)	Cav3.3	[276]
Imperatoxin A	*Pandinus imperator*	33 (3)	RyR1, RyR2, RyR3	[276]
Intrepicalcin	*Thorellius intrepidus*	33 (3)	RyR1	[277]
Urocalcin	*Urodacus yaschenkoi*	33 (3)	RyR1	[278]
Vejocalcin	*Vaejovis mexicanus*	33 (3)	RyR1	[278]
Hemicalcin	*Hemiscorpius lepturus*	33 (3)	RyR1	[279]
Hadrucalcin	*Hoffmannihadrurus gertschi*	35 (3)	RyR1, RyR2	[273]
φ-LITX-Lw1a	*Hormurus waigiensis*	36 (2)	RyR1, RyR2	[272]
Maurocalcin	*Scorpio palmatus*	33 (3)	RyR1	[280,281]
Opicalcin 1	*Opistophthalmus carinatus*	33 (3)	RyR1	[278]
Opicalcin 2	*Opistophthalmus carinatus*	33 (3)	RyR1	[278]

**Table 5:** Representative scorpion toxins targeting chloride channels (ClTx).

ClTx	Scorpion Species	Peptide Length (S–S Bridge)	Targets	References
Ammp2	*Androctonus mauritanicus*	35 (3)	ClC-3, MMP2	[299]
I5A	*Mesobuthus eupeus*	35 (4)	ClC-3, MMP2	[303]
Chlorotoxin	*Leiurus quinquestriatus*	36 (4)	ClC-3, MMP2	[300,301,302]
Lqh7-1	*Leuirus quinquestratus*	34 (4)	TMEM16A/Ano1	[304]
Lqh2-2	*Leuirus quinquestratus*	34 (4)	TMEM16A/Ano1	[304]
BmKCT	*Olivierus martensii*	35 (4)	ClC-3, MMP2	[305]
Bs14	*Hottentotta tamulus*	36 (4)	ClC-3, MMP2	[313]
PBITX1	*Parabuthus schlechteri*	25 (4)	ClC-3, MMP2	[307]

**Table 6:** Representative scorpion toxins targeting TRP channels.

TRPTx	Scorpion Species	Peptide Length (S–S Bridge)	Targets	References
BmP01	*Olivierus martensii*	29 (3)	Kv1.1, TRPV1	[322,325]
WaTx	*Urodacus manicatus*	33 (2)	TRPA1	[324,327]
Tx203	*Buthus israelis*	38 (3)	TRPV1	[322]
OdK1	*Odontobuthus doriae*	29 (3)	Kv1.2, TRPV1	[328]

## Data Availability

No new data were created or analyzed in this study.
